# Synthesis and Identification of Novel Berberine Derivatives as Potent Inhibitors against TNF-α-Induced NF-κB Activation

**DOI:** 10.3390/molecules22081257

**Published:** 2017-07-27

**Authors:** Yan-Xiang Wang, Lu Liu, Qing-Xuan Zeng, Tian-Yun Fan, Jian-Dong Jiang, Hong-Bin Deng, Dan-Qing Song

**Affiliations:** Institute of Medicinal Biotechnology, Chinese Academy of Medical Science and Peking Union Medical College, Beijing 100050, China; wangyanxiang@imb.pumc.edu.cn (Y.-X.W.); liulu208@163.com (L.L.); zqx50810793@163.com (Q.-X.Z.); fty1668@163.com (T.-Y.F.); jiang.jdong@163.com (J.-D.J.)

**Keywords:** berberine, anti-inflammatory, NF-κB, IκB kinase, structure-activity relationship

## Abstract

Twenty-three new berberine (BBR) analogues defined on substituents of ring D were synthesized and evaluated for their activity for suppression of tumor necrosis factor (TNF)-α-induced nuclear factor (NF)-κB activation. Structure–activity relationship (SAR) analysis indicated that suitable tertiary/quaternary carbon substitutions at the 9-position or rigid fragment at position 10 might be beneficial for enhancing their anti-inflammatory potency. Among them, compounds **2d**, **2e**, **2i** and **2j** exhibited satisfactory inhibitory potency against NF-κB activation, with an inhibitory rate of around 90% (5 μM), much better than BBR. A preliminary mechanism study revealed that all of them could inhibit TNF-α-induced NF-κB activation via impairing IκB kinase (IKK) phosphorylation as well as cytokines interleukin (IL)-6 and IL-8 induced by TNF-α. Therefore, the results provided powerful information on further structural modifications and development of BBR derivatives into a new class of anti-inflammatory candidates for the treatment of inflammatory diseases.

## 1. Introduction

Inflammation represents a response to tissue injury induced by a wide variety of stimuli, such as pathogens, damaged cells, or irritants, and is a protective response involving immune cells, blood vessels, and molecular mediators [1,2,3,4]. As a consequence, diverse pathological conditions involve inflammatory processes including arthritis, atherosclerosis, the metabolic syndrome, sepsis, and cancer. For most of these conditions, no satisfactory treatment is available [5,6]. Initial stages of inflammation involve cytokine-mediated activation of the vascular endothelium leading to adhesion and transmigration of leukocytes into the site of inflammation. Many of the pro-inflammatory processes elicited at the endothelium and leukocytes are mediated by the transcription factor nuclear factor (NF)-κB. The most prevalent inducers of the NF-κB signaling pathway are cytokines such as tumor necrosis factor (TNF)-α and interleukin (IL)-1, various mitogens, and bacterial components such as lipopolysaccharides (LPS).

NF-κB is the name used for a family of homodimers and heterodimers, the NF-κB dimers are maintained in an inactive state in the cytoplasm bound to the inhibitor of κB (IκB) proteins, of which the prototypical member is IκB-α [7]. Upon a pro-inflammatory signal, such as binding of TNF-α to its membrane receptor, IκB-α becomes phosphorylated at Ser^32^/Ser^36^ by IκB kinase (IKK). The IKK is a multi-subunit kinase complex, most typically composed of IKK-α and IKK-β and two molecules of IKKγ/NF-kappa-B essential modulator (NEMO) [8]. The IKK-catalyzed phosphorylation triggers degradation of IκB-α leading to the release of NF-κB followed by its translocation to the nucleus where it regulates gene expression [9]. As NF-κB is a key regulator of many pro-inflammatory responses, inhibition of different mediators of the NF-κB signaling pathway, including IKK, has emerged as a promising approach for the development of anti-inflammatory candidates.

**Figure 1:**
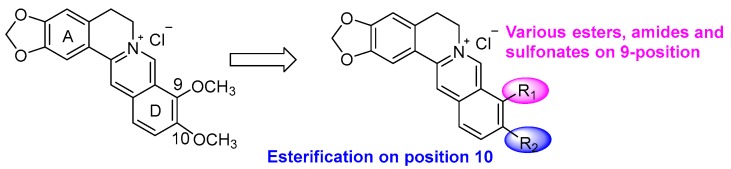
Chemical structures of berberine (BBR), and its structure modification strategy.

Natural isoquinoline alkaloid berberine (BBR, Figure 1), as a nonprescription anti-diarrhea drug, has been extensively used in China for decades with a confirmed safety. Recently, several other pharmacological and biological properties of BBR including anti-inflammatory and anti-carcinogenic activities have been identified [10,11,12,13,14,15,16]. It was reported that BBR could suppress NF-κB activation induced by various inflammatory agents or carcinogens with a mild potency [17]. In our study, BBR’s moderate anti-inflammatory potency was further confirmed by an inhibitory rate of 36% at the concentration of 5 μM. The unique isoquinoline skeleton of BBR provoked us to conduct structural modification and optimization to enhance the anti-inflammatory effect. Therefore, in our present study, taking BBR as the lead, a series of novel BBR analogues defined on the substituents of ring D (Figure 1) was achieved to elucidate the structure-activity relationship (SAR) and develop a novel class of anti-inflammatory agents. Specifically, novel ester, amide, and sulfonate BBR derivatives were prepared and evaluated for their effect to inhibit TNF-α-induced NF-κB activation, and the mechanism exploration of the key compounds was carried out as well.

## 2. Results and Discussion

### 2.1. Chemistry

**Scheme 1:**
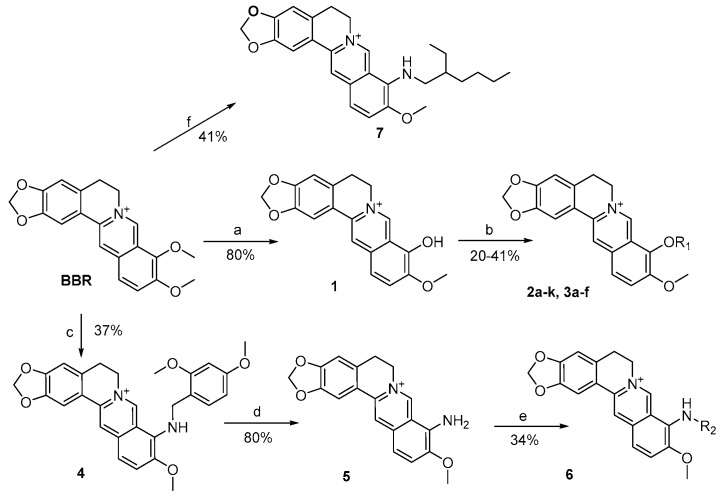
Reagents and conditions: (a) 195 °C, 30–40 mmHg, 60 min; (b) R_1_COX/R_1_SO_2_Cl, triethylamine, CH_3_CN, reflux; (c) 2,4-Dimethoxybenzylamine, 120 °C; (d) HCl, CH_3_OH; (e) R_2_COX, pyridine, CH_2_Cl_2_, reflux; (f) R-NH_2_, 120 °C.

Firstly, taking commercial available BBR as the starting material, a demethylation reaction was conducted to afford the key intermediate **1** (Scheme 1) [18]. The esters **2a**–**k** and sulfonates **3a**–**f** were obtained by esterification and sulfonation of compound **1** with various acyl chloride and sulfonyl chloride, respectively, using acetonitrile as solvent and triethylamine as the base. Compounds **4** and **7** were prepared in the presence of corresponding amino compounds [19]. After acidification, compound **6** was obtained taking pyridine as the base.

**Scheme 2:**
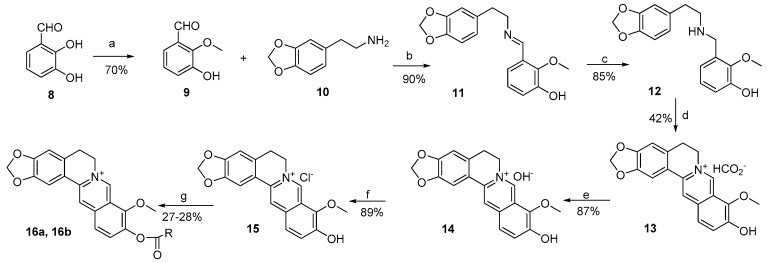
Reagents and conditions: (a) CH_3_I, room temperature (r.t.); (b) 100 °C, 8 h; (c) NaBH_4_, methanol, reflux, 5 h; (d) glyoxal, formic acid, CuSO_4_, HCl, 100 °C, 5 h; (e) methanol/H_2_O, CaO, r.t., 2 h; (f) ethanol, HCl, r.t., 0.5 h; (g) RCOX, triethylamine, CH_3_CN, reflux.

Secondly, compounds **16a** and **16b** were synthesized through a seven-step process (Scheme 2), using commercially available 2,3-dihydroxybenzaldehyde (**8**) as the starting materials with the methods reported previously [20,21]. Condensation was conducted between 2-methoxy-3-hydroxy benzaldehyde (**9**) and homopiperonylamine (**10**) after selective methylation of **8**. In the intermolecular cyclization of the intermediate **12**, the skeleton formed according to Pictet-Spengler cyclization and Friedel-Crafts alkylation rule in one step, and, subsequently, the key intermediate **13** was obtained with an ideal yield of 53%. After acidification, alkalization, and esterification, the final products **16a** and **16b** were purified via flash column chromatography using methanol/dichloromethane as the gradient eluent.

### 2.2. Biology

#### 2.2.1. SAR for Suppressing TNF-α-induced NF-κB Activation

All the newly synthesized compounds were examined in 293T cells for their anti-inflammatory activities. Given the key role of NF-κB signaling in inflammation, we investigated these analogues for their anti-inflammatory abilities by using TNF-α-induced NF-κB-responsive promoter reporter assays. Structures of the analogues and their inhibitory rates on TNF-α-induced NF-κB-responsive promoter activity are shown in Table 1.

SAR analysis was first focused on the influence of substitutions on position 9 of ring D, by which eleven new ester derivatives (**2a**–**k**) were prepared and tested taking PS1145 as the positive control [22]. Different kinds of cyclic carboxylic acid were introduced on 9-hydroxyl by which cyclopropanecarboxylate (**2a**), cyclopentanecarboxylate (**2b**), 2-cyclopentylacetate (**2c**), and 1-methylcyclohexane-1-carboxylate (**2d**) derivatives were examined for their abilities to inhibit NF-κB activation. An inspiring potency with the inhibitory rate of 96% was given by compound **2d** while compounds **2a**–**c** only gave comparable inhibitory rates (34–56%) to BBR (36%). Then, two bridged-ring derivatives (**2e**,**f**) were created; norbornane substituted compound **2e** exhibited an improved inhibitory effect on NF-κB activation with the rate of 83%, while the activity of chlorinated adamantane compound **2f** was only comparable to that of BBR. Finally, three alkyl ring-opening analogues bearing tertiary carbon and quaternary carbon substitutions (**2g**–**i**) were prepared and tested, and they gave the improved activities with inhibitory rates of 81–96%. Based on the results above, it was speculated that the introduction of an ester group bearing a suitable tertiary/quaternary carbon substitution at the 9-position was beneficial for the activity. Moreover, the activity was retained when a benzene or unsaturated heterocycle ring (**2j**,**k**) was inserted between the ester bond and quaternary carbon substituent.

Meanwhile, 6 sulfonate derivatives (**3a**–**f**) were designed to explore further SAR on position 9. Compounds **3a**–**d**, possessing substituted benzenesulfonate, were tested, and they could suppress NF-κB expression by 65–87%. It seemed that trifluoromethyl was a more favorable substitution than a nitro group for the ability to inhibit TNF-α-induced NF-κB activation. Then, 10′-camphorsulfonyl analogues **3e**,**f** with different chiral configurations were generated, and apparently, compound **3e** with a d-configuration showed obviously higher activity than its entantiomer **3f**, which indicated the possible effect of chiral configuration.

Then, converting the sulfonyl linker to an amine or amide linker, and the generated compounds **4**–**7** did not show improved activities compared to the lead BBR, and the result might hint that amine and amide were not suitable to be applied as linkers for the inhibitory activity.

Next, the SAR study was conducted for the substituents on the 10-position of ring D. Introducing adamantate at position 10 in BBR, compounds **16a** and **16b** gave satisfactory potencies with inhibitory rates of 96% and 84%, respectively, which indicated that rigid structure on position 10 might be also favorable for the ability to inhibit TNF-α-induced NF-κB activation.

**Figure 2:**
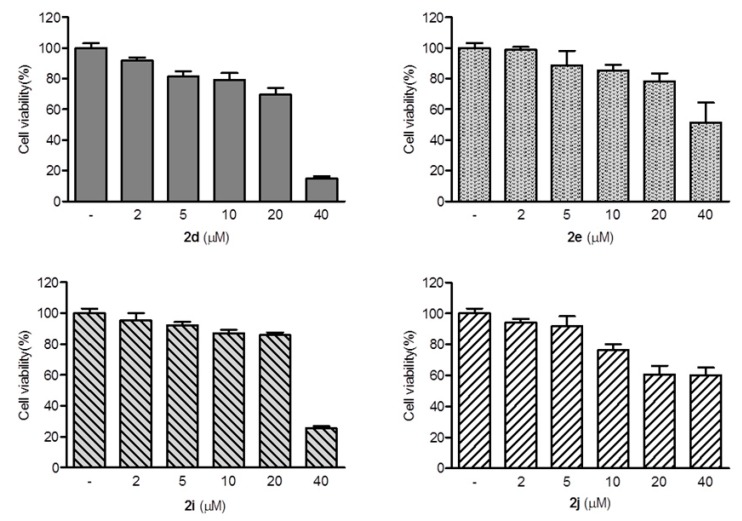
Cytotoxic effects of compounds **2d**, **2e**, **2i** and **2j** on 293T cells. Following pretreatment with compounds **2d**, **2e**, **2i** and **2j** at the indicated concentrations for 24 h, the cell viability of 293T cells were determined by MTT assay.

Based on the preliminary SAR analysis, the IC_50_ of representative compounds **2d**, **2e**, **2i** and **2j** on inhibition of TNF-α-induced NF-κB activation were tested, as listed in Table 2. To evaluate the effect of the novel BBR derivatives on cell viability, the cytotoxic effects of representative compounds **2d**, **2e**, **2i** and **2j** on 293T cells were determined by MTT assay. Our results revealed that **2d**, **2e**, **2i** and **2j** failed to affect cell viability for 24 h at concentrations up to 20 μM (Figure 2). These data demonstrated that **2d**, **2e**, **2i** and **2j** within 20 μM has little cytotoxic effects on 293T cells.

#### 2.2.2. Preliminary Mechanism Study

**Figure 3:**
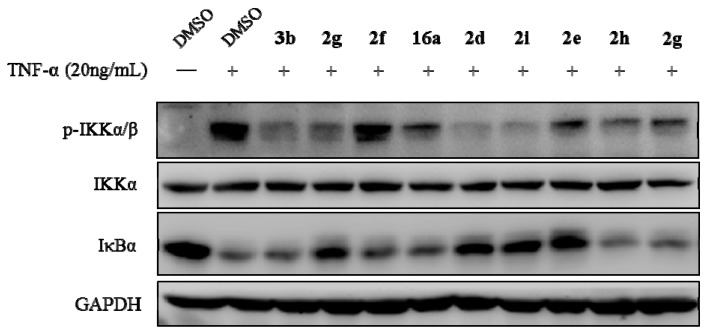
BBR analogues inhibited TNF-α-induced NF-κB activation by impairing IκB kinase (IKK) phosphorylation. Following pretreatment with BBR analogs **2d**–**j**, **3b** and **16a** (10 μM) for 2 h, 293T cells were treated with TNF-α (20 ng/mL) for 30 min. The phosphorylation of IKKα/β (Ser180/181) and expression levels of IKKα and IκBα were measured by Western blot using corresponding antibodies. GADPH = glyceraldehyde-3-phosphate dehydrogenase.

**Figure 4:**
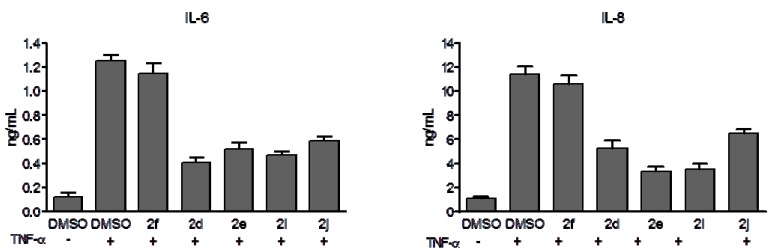
Effects of BBR analogues on TNF-α-induced cytokine of interleukin (IL)-6 and IL-8. Following pretreatment with compounds **2d**, **2e**, **2f**, **2i** and **2j** (10 μM) for 2 h, 293T cells were treated with TNF-α (20 ng/mL) for 6 h, and then the medium was collected for ELISA analysis.

Nine key compounds (**2d**–**j**, **3b** and **16a**) with different types of structure were selected to investigate the preliminary mechanism of NF-κB inactivation. Considering that IKK plays a critical role in TNF-α-induced NF-κB activation, the experiment was carried out to verify if the 9 compounds suppressed NF-κB activation through the IKK pathway. The translocation of NF-κB to the nucleus is preceded by the phosphorylation, ubiquitination, and proteolytic degradation of IκBα [23]. To determine whether inhibition of TNF-α-induced NF-κB activation was due to inhibition of IκBα degradation through IKK, we pretreated 293T cells with our representative compounds and then exposed them to TNF-α for 2 h. We then examined the cells for IKK phosphorylation with antibodies specific for phospho-IKKα (Ser180), phosphor-IKKβ (Ser181), and IκBα degradation by Western blot. As shown in Figure 3, TNF-α induced IKKα/β phosphorylation continued to increase at 120 min but had no effect on compounds **2d**, **2e**, **2h**, **2i**, **2j** and **16a**-pretreated cells, this was not the result of the variation of IKK expression, as the total amount of IKK protein remained unchanged during the treatment (Figure 3). In addition, compounds **2d**, **2e**, **2i** and **2j** delayed TNF-α-induced degradation of IκBα (Figure 3). Moreover, **2d**, **2e**, **2i**, **2j**, as well as the negative control **2f** inhibited TNF-α-induced expression of cytokines including IL-6 and IL-8, as depicted in Figure 4. These results demonstrated that compounds **2d**, **2e**, **2i** and **2j** inhibited both TNF-α-induced IKK phosphorylation and IκBα degradation, indicating these compounds had great potential as anti-inflammatory agents. IKK serves as a key node in inflammatory signaling, inhibition of which may represent a novel therapeutic target for inflammatory diseases such as rheumatoid arthritis and atherosclerosis. Identification of compounds **2d**, **2e**, **2i** and **2j** as novel IKK inhibitors will allow us to better define the potential of using BBR derivatives for the treatment of inflammatory diseases.

## 3. Materials and Methods

### 3.1. Apparatus, Materials, and Analysis Reagents

Antibodies against IκBα, IKKα, phospho-IKKα/β (Ser180/181), and glyceraldehyde-3-phosphate dehydrogenase (GADPH) were purchased from Cell Signaling Technology (Danvers, MA, USA). TNF-α was obtained from R&D Systems (Minneapolis, MN, USA). The p5×nuclear factor (NF)-κB-luciferase reporter plasmid, the pRL-TK plasmid and Dual-luciferase reporter assay system were from Promega (Madison, WI, USA).

Melting point (mp) was obtained with CXM-300 melting point apparatus and was uncorrected. The ^1^H-NMR spectra was performed on a Varian Inova 500 or 600 MHz spectrometer (Varian, San Francisco, CA, USA) and ^13^C-NMR on a Bruker Avance III 500 or 600 spectrometer in Dimethyl sulfoxide (DMSO)-*d*_6_, or CDCl_3_, with Me_4_Si as the internal standard. Electrospray ionization (ESI) high-resolution mass spectra (HRMS) were recorded on an Autospec UItima-TOF mass spectrometer (Micromass UK Ltd., Manchester, UK). Flash chromatography was performed on a Combiflash Rf 200 (Teledyne, NE, USA), particle size 0.038 mm.

### 3.2. Synthesis

#### 3.2.1. General Procedure for the Synthesis of **2a**–**k** and **3a**–**f**

BBR (3.71 g, 10 mmol) was heated at 195–210 °C for 10–15 min under vacuum (30–40 mmHg) to afford the black oil, which was acidified with ethanol/concentrated HCl (95:5). The solvent was removed by evaporation and the residue was collected and then purified by flash chromatography over silica gel using CH_2_Cl_2_/CH_3_OH as the gradient eluent, giving the title compound **1** (2.85 g, 80%) as an orange solid.

To a stirred solution of **1** (100 mg, 0.28 mmol) in anhydrous CH_3_CN, triethylamine (175 µL, 1.26 mmol) was added and heated to 70 °C. Then the RCOX/RSO_2_Cl (1.1–1.2 eq) was added and stirred for 5–6 h. The mixture was cooled to precipitate completely, filtrated and washed with CH_2_Cl_2_ to afford target compounds **2a**–**k** and **3a**–**f**.

*2,3-Methylenedioxy-9-((cyclopropanecarbonyl)oxy)-10-methoxy* *protoberberine chloride* (**2a**). Compound **1** (100 mg, 0.28 mmol) was treated with cyclopropanecarbonyl chloride (28 µL, 0.31 mmol) according to the general procedure to give the desired product **2a** as a yellow solid, yield: 34%; M.p.: 193–195 °C (Dec.); ^1^H-NMR (500 MHz, DMSO-*d*_6_) δ 9.89 (s, 1H, CH_arom_), 9.08 (s, 1H, CH_arom_), 8.29 (d, *J* = 9.2 Hz, 1H, CH_arom_), 8.21 (d, *J* = 9.2 Hz, 1H, CH_arom_), 7.82 (s, 1H, CH_arom_), 7.11 (s, 1H, CH_arom_), 6.19 (s, 2H, OCH_2_O), 4.98 (t, *J* = 6.4 Hz, 2H, CH_2_), 4.04 (s, 3H, OCH_3_), 3.22 (t, *J* = 6.4 Hz, 2H, CH_2_), 2.13 (tt, *J* = 7.8, 4.7 Hz, 1H, CH), 1.26–1.16 (m, 4H, 2 × CH_2_); ^13^C-NMR (126 MHz, DMSO-*d*_6_) δ 172.3, 151.0, 150.6, 148.3, 144.9, 138.7, 134.2, 133.5, 131.5, 127.3, 126.5, 121.8, 121.3, 121.0, 109.1, 106.2, 102.8, 57.9, 56.7, 26.8, 13.4, 10.4(2); HRMS: calcd. for C_23_H_20_NO_5_Cl [M − Cl]^+^ 390.1336, found 390.1342.

*2,3-Methylenedioxy-9-((cyclopentanecarbonyl)oxy)-10-methoxy* *protoberberine chloride* (**2b**). Compound **1** (100 mg, 0.28 mmol) was treated with cyclopentanecarbonyl chloride (38 µL, 0.31 mmol) according to the general procedure to give the desired product **2b** as a yellow solid, yield: 32%; M.p.: 207–209 °C (Dec.); ^1^H-NMR (500 MHz, DMSO-*d*_6_) δ 9.89 (s, 1H, CH_arom_), 9.07 (s, 1H, CH_arom_), 8.29 (d, *J* = 9.2 Hz, 1H, CH_arom_), 8.21 (d, *J* = 9.2 Hz, 1H, CH_arom_), 7.83 (s, 1H, CH_arom_), 7.11 (s, 1H, CH_arom_), 6.19 (s, 2H, OCH_2_O), 4.96 (t, *J* = 6.3 Hz, 2H, CH_2_), 4.03 (s, 3H, OCH_3_), 3.47–3.38 (m, 1H, CH), 3.23 (t, *J* = 6.3 Hz, 2H, CH_2_), 2.14–2.00 (m, 4H, 2 × CH_2_), 1.79–1.64 (m, 4H, 2 × CH_2_); ^13^C-NMR (126 MHz, DMSO-*d*_6_) δ 174.1, 150.9, 150.6, 148.3, 145.1, 138.7, 134.5, 133.5, 131.5, 127.2, 126.5, 121.8, 121.2, 121.0, 109.1, 106.2, 102.8, 57.9, 56.0, 43.6, 30.1(2), 26.8, 26.1(2); HRMS: calcd. for C_25_H_24_NO_5_Cl [M − Cl]^+^ 418.1649, found 418.1653.

*2,3-Methylenedioxy-9-(2′-cyclopentylacetoxy)-10-methoxy* *protoberberine chloride* (**2c**). Compound **1** (100 mg, 0.28 mmol) was treated with 2-cyclopentylacetyl chloride (42 µL, 0.31 mmol) according to the general procedure to give the desired product **2c** as a yellow solid, yield: 38%; M.p.: 186–188 °C (Dec.); ^1^H-NMR (500 MHz, DMSO-*d*_6_) δ 9.94 (s, 1H, CH_arom_), 9.06 (s, 1H, CH_arom_), 8.29 (d, *J* = 9.2 Hz, 1H, CH_arom_), 8.21 (d, *J* = 9.2 Hz, 1H, CH_arom_), 7.82 (s, 1H, CH_arom_), 7.11 (s, 1H, CH_arom_), 6.19 (s, 2H, OCH_2_O), 4.95 (t, *J* = 6.3 Hz, 2H, CH_2_), 4.03 (s, 3H, OCH_3_), 3.23 (t, *J* = 6.3 Hz, 2H, CH_2_), 2.88 (d, *J* = 7.3 Hz, 2H, CH_2_), 2.41–2.30 (m, 1H, CH), 1.96–1.88 (m, 2H, CH_2_), 1.74–1.63 (m, 2H, CH_2_), 1.63–1.53 (m, 2H, CH_2_), 1.40–1.27 (m, 2H, CH_2_); ^13^C-NMR (126 MHz, CDCl_3_) δ 171.5, 151.3, 151.0, 148.7, 147.3, 138.3, 136.3, 133.1, 131.0, 125.9, 125.5, 122.5, 120.2, 119.7, 108.9, 105.3, 102.4, 57.2, 55.3, 40.5, 36.7, 32.6(2), 27.8, 25.4(2); HRMS: calcd. for C_26_H_26_NO_5_Cl [M − Cl]^+^ 432.1805, found 432.1808.

*2,3-Methylenedioxy-9-((1′-methylcyclohexane-1′-carbonyl)oxy)-10-methoxy* *protoberberine chloride* (**2d**). Compound **1** (100 mg, 0.28 mmol) was treated with 1-methyl-1-cyclohexanecarboxylic acid (48 mg, 0.34 mmol) according to the general procedure to give the desired product **2d** as a yellow solid, yield: 39%; M.p.: 215–217 °C (Dec.); ^1^H-NMR (500 MHz, DMSO-*d*_6_) δ 9.42 (s, 1H, CH_arom_), 9.09 (s, 1H, CH_arom_), 8.30 (d, *J* = 9.2 Hz, 1H, CH_arom_), 8.22 (d, *J* = 9.2 Hz, 1H, CH_arom_), 7.84 (s, 1H, CH_arom_), 7.12 (s, 1H, CH_arom_), 6.19 (s, 2H, OCH_2_O), 4.96 (t, *J* = 6.2 Hz, 2H, CH_2_), 4.03 (s, 3H, OCH_3_), 3.21 (t, *J* = 6.2 Hz, 2H, CH_2_), 2.24–2.15 (m, 2H, CH_2_), 1.70–1.34 (m, 11H, 4 × CH_2_ and CH_3_); ^13^C-NMR (126 MHz, DMSO-*d*_6_) δ 175.1, 150.8, 150.6, 148.4, 144.4, 138.7, 134.4, 133.7, 131.6, 127.3, 126.6, 121.5, 121.4, 121.0, 109.1, 106.2, 102.8, 57.8, 56.5, 44.0, 35.6(2), 26.8, 26.3, 25.9, 23.1(2); HRMS: calcd. for C_27_H_28_NO_5_Cl [M − Cl]^+^ 446.1962, found 446.1962.

*2,3-Methylenedioxy-9′-(2-(bicyclo[2.2.1]heptan-2-yl)acetoxy)-10-methoxy protoberberine chloride* (**2e**). Compound **1** (100 mg, 0.28 mmol) was treated with 2-norbornaneacetic acid (49 µL, 0.34 mmol) according to the general procedure to give the desired product **2e** as a yellow solid, yield: 24%; M.p.: 189–191 °C (Dec.); ^1^H-NMR (500 MHz, DMSO-*d*_6_) δ 10.01–9.94 (m, 1H, CH_arom_), 9.07 (s, 1H, CH_arom_), 8.29 (d, *J* = 9.2 Hz, 1H, CH_arom_), 8.21 (d, *J* = 9.2 Hz, 1H, CH_arom_), 7.82 (s, 1H, CH_arom_), 7.11 (s, 1H, CH_arom_), 6.19 (s, 2H, OCH_2_O), 4.96 (t, *J* = 6.3 Hz, 2H, CH_2_), 4.03 (s, 3H, OCH_3_), 3.23 (t, *J* = 6.3 Hz, 2H, CH_2_), 2.83 (dd, *J* = 16.0, 7.5 Hz, 1H, CH_2_), 2.72 (dd, *J* = 16.0, 7.5 Hz, 1H, CH_2_), 2.29–2.24 (m, 1H, CH), 2.18–2.14 (m, 1H, CH_2_), 2.05–1.97 (m, 1H, CH_2_), 1.62–1.44 (m, 4H, 2 × CH_2_), 1.29–1.12 (m, 4H, 2 × CH_2_); ^13^C-NMR (126 MHz, DMSO-*d*_6_) δ 170.5, 151.0, 150.6, 148.3, 145.1, 138.7, 134.2, 133.5, 131.5, 127.3, 126.5, 121.8, 121.2, 121.0, 109.1, 106.2, 102.8, 57.8, 55.9, 41.2, 40.8, 38.6, 38.0, 36.9, 35.5, 30.1, 28.9, 26.8; HRMS: calcd. for C_28_H_28_NO_5_Cl [M − Cl]^+^ 458.1962, found 458.1963.

*2,3-Methylenedioxy-9-((3′-chloroadamantane-1′-carbonyl)oxy)-10-methoxy* *protoberberine chloride* (**2f**). Compound **1** (100 mg, 0.28 mmol) was treated with 3-chloroadamantane-1-carboxylic acid (73 mg, 0.34 mmol) according to the general procedure to give the desired product **2f** as a brown solid, yield: 33%; M.p.: 206–208 °C (Dec.); ^1^H-NMR (500 MHz, DMSO-*d*_6_) δ 9.63 (s, 1H, CH_arom_), 9.08 (s, 1H, CH_arom_), 8.29 (d, *J* = 9.2 Hz, 1H, CH_arom_), 8.22 (d, *J* = 9.2 Hz, 1H, CH_arom_), 7.83 (s, 1H, CH_arom_), 7.12 (s, 1H, CH_arom_), 6.19 (s, 2H, OCH_2_O), 4.99 (t, *J* = 6.3 Hz, 2H, CH_2_), 4.03 (s, 3H, OCH_3_), 3.23 (t, *J* = 6.3 Hz, 2H, CH_2_), 2.56 (s, 2H CH_2_), 2.38–2.31 (m, 2H, CH_2_), 2.21–2.17 (m, 4H, 2 × CH_2_), 2.17–2.08 (m, 4H, 2 × CH_2_), 1.79–1.67 (m, 2H, 2 × CH); ^13^C-NMR (126 MHz, DMSO-*d*_6_) δ 173.2, 150.6, 150.6, 148.4, 144.6, 138.7, 134.3, 133.6, 131.56, 127.4, 126.5, 121.5, 121.4, 121.0, 109.1, 106.2, 102.8, 69.1, 58.0, 56.2, 48.0, 46.7(2), 45.4, 37.2(2), 34.3, 31.2(2), 26.8; HRMS: calcd. for C_30_H_29_NO_5_Cl_2_ [M − Cl]^+^ 518.1728, found 518.1733.

*2,3-Methylenedioxy-9-pivaloyloxy-10-methoxy* *protoberberine chloride* (**2g**). Compound **1** (100 mg, 0.28 mmol) was treated with pivaloyl chloride (38 µL, 0.31 mmol) according to the general procedure to give the desired product **2g** as a yellow solid, yield: 32%; M.p.: 205–207 °C (Dec.); ^1^H-NMR (500 MHz, DMSO-*d*_6_) δ 9.58 (s, 1H, CH_arom_), 9.09 (s, 1H, CH_arom_), 8.29 (d, *J* = 9.2 Hz, 1H, CH_arom_), 8.22 (d, *J* = 9.2 Hz, 1H, CH_arom_), 7.83 (s, 1H, CH_arom_), 7.12 (s, 1H, CH_arom_), 6.19 (s, 2H, OCH_2_O), 4.99 (t, *J* = 6.3 Hz, 2H, CH_2_), 4.03 (s, 3H, OCH_3_), 3.22 (t, *J* = 6.3 Hz, 2H, CH_2_), 1.47 (s, 9H,(CH_3_)_3_); ^13^C-NMR (151 MHz, CD_3_OD) δ 175.7, 150.9, 150.9, 148.5, 143.4, 138.8, 134.7, 133.7, 130.6, 126.4, 125.5, 121.7, 120.7, 120.3, 107.9, 105.2, 102.3, 56.3, 56.1, 39.3, 26.6, 26.2(3); HRMS: calcd. for C_24_H_24_NO_5_Cl [M − Cl]^+^ 406.1649, found 406.1653.

*2,3-Methylenedioxy-9-((3′,3′-dimethylbutanoyl)oxy)-10-methoxy* *protoberberine chloride* (**2h**). Compound **1** (100 mg, 0.28 mmol) was treated with 3,3-dimethylbutyryl chloride (43 µL, 0.31 mmol) according to the general procedure to give the desired product **2h** as a yellow solid, yield: 27%; M.p.: 212–214 °C (Dec.); ^1^H-NMR (500 MHz, DMSO-*d*_6_) δ 9.96 (s, 1H, CH_arom_), 9.07 (s, 1H, CH_arom_), 8.30 (d, *J* = 9.2 Hz, 1H, CH_arom_), 8.22 (d, *J* = 9.2 Hz, 1H, CH_arom_), 7.83 (s, 1H, CH_arom_), 7.11 (s, 1H, CH_arom_), 6.19 (s, 2H, OCH_2_O), 4.96 (t, *J* = 6.3 Hz, 2H, CH_2_), 4.04 (s, 3H, OCH_3_), 3.23 (t, *J* = 6.3 Hz, 2H, CH_2_), 2.75 (s, 2H, CH_2_), 1.18 (s, 9H, (CH_3_)_3_); ^13^C-NMR (126 MHz, DMSO-*d*_6_) δ 169.5, 151.0, 150.6, 148.3, 145.1, 138.7, 134.1, 133.6, 131.5, 127.3, 126.5, 121.8, 121.2, 121.0, 109.1, 106.2, 102.8, 57.7, 55.9, 47.6, 31.4, 30.0(3), 26.8; HRMS: calcd. for C_25_H_26_NO_5_Cl [M − Cl]^+^ 420.1805, found 420.1813.

*2,3-Methylenedioxy-9-((2′-propylpentanoyl)oxy)-10-methoxy* *protoberberine chloride* (**2i**). Compound **1** (100 mg, 0.28 mmol) was treated with 2-propylpentanoyl chloride (58 µL, 0.34 mmol) according to the general procedure to give the desired product **2i** as a yellow solid, yield: 24%; M.p.: 199–201 °C (Dec.); ^1^H-NMR (500 MHz, DMSO-*d*_6_) δ 9.68 (s, 1H, CH_arom_), 9.10 (s, 1H, CH_arom_), 8.30 (d, *J* = 9.2 Hz, 1H, CH_arom_), 8.23 (d, *J* = 9.2 Hz, 1H, CH_arom_), 7.83 (s, 1H, CH_arom_), 7.11 (s, 1H, CH_arom_), 6.19 (s, 2H, OCH_2_O), 4.95 (t, *J* = 6.3 Hz, 2H, CH_2_), 4.02 (s, 3H, OCH_3_), 3.23 (t, *J* = 6.3 Hz, 2H, CH_2_), 2.95 (p, *J* = 6.6 Hz, 1H, CH), 1.88–1.79 (m, 2H, CH_2_), 1.72–1.63 (m, 2H, CH_2_), 1.56–1.42 (m, 4H, 2 × CH_2_), 0.99 (t, *J* = 7.3 Hz, 6H, 2 × CH_3_); ^13^C-NMR (126 MHz, DMSO-*d*_6_) δ 173.4, 150.9, 150.6, 148.4, 144.6, 138.7, 134.1, 133.6, 131.5, 127.3, 126.5, 121.6, 121.3, 121.0, 109.1, 106.2, 102.8, 57.8, 56.2, 44.4, 33.6(2), 26.8, 20.3(2), 14.7(2); HRMS: calcd. for C_27_H_30_NO_5_Cl [M − Cl]^+^ 448.2118, found 448.2128.

*2,3-Methylenedioxy-9-((p-tert-butylbenzoyl)oxy)-10-methoxy* *protoberberine chloride* (**2j**). Compound **1** (100 mg, 0.28 mmol) was treated with 4-tert-butylbenzoyl chloride (61 µL, 0.31 mmol) according to the general procedure to give the desired product **2j** as a brown solid, yield: 28%; M.p.: 205–207 °C (Dec.); ^1^H-NMR (500 MHz, DMSO-*d*_6_) δ 10.00 (s, 1H, CH_arom_), 9.11 (s, 1H, CH_arom_), 8.35 (d, *J* = 9.2 Hz, 1H, , CH_arom_), 8.27 (d, *J* = 9.2 Hz, 1H, CH_arom_), 8.20 (d, *J* = 8.5 Hz, 2H, 2 × CH_arom_), 7.85 (s, 1H, CH_arom_), 7.72 (d, *J* = 8.5 Hz, 2H, 2 × CH_arom_), 7.10 (s, 1H, CH_arom_), 6.19 (s, 2H, OCH_2_O), 4.91 (t, *J* = 6.3 Hz, 2H, CH_2_), 4.03 (s, 3H, OCH_3_), 3.20 (t, *J* = 6.3 Hz, 2H, CH_2_), 1.38 (s, 9H, (CH_3_)_3_); ^13^C-NMR (126 MHz, DMSO-*d*_6_) δ 164.0, 158.6, 151.1, 150.6, 148.4, 145.1, 138.8, 134.3, 133.6, 131.5(2), 131.1, 127.5, 126.6(2), 126.5, 125.9, 122.0, 121.3, 121.0, 109.1, 106.2, 102.8, 57.9, 55.8, 35.8, 31.5(3), 26.8; HRMS: calcd. for C_30_H_28_NO_5_Cl [M − Cl]^+^ 482.1962, found 482.1962.

*2,3-Methylenedioxy-9-((3′-tert-butyl-1′-methyl-1H-pyrazole-5′-carbonyl)oxy)-10-methoxy protoberberine chloride* (**2k**). Compound **1** (100 mg, 0.28 mmol) was treated with 5-tert-butyl-2-methylpyrazole-3-carbonyl chloride (59 µL, 0.34 mmol) according to the general procedure to give the desired product **2k** as a yellow solid, yield: 42%; M.p.: 207–209 °C (Dec.); ^1^H-NMR (500 MHz, DMSO-*d*_6_) δ 10.03 (s, 1H, CH_arom_), 9.12 (s, 1H, CH_arom_), 8.36 (d, *J* = 9.2 Hz, 1H, CH_arom_), 8.29 (d, *J* = 9.2 Hz, 1H, CH_arom_), 7.84 (s, 1H, CH_arom_), 7.15 (s, 1H, CH_pyrazole_), 7.11 (s, 1H, CH_arom_), 6.19 (s, 2H, OCH_2_O), 4.92 (t, *J* = 6.4 Hz, 2H, CH_2_), 4.13 (s, 3H, NCH_3_), 4.06 (s, 3H, OCH_3_), 3.22 (t, *J* = 6.4 Hz, 2H, CH_2_), 1.34 (s, 9H, (CH_3_)_3_); ^13^C-NMR (126 MHz, DMSO-*d*_6_) δ 160.5, 157.2, 151.1, 150.7, 148.4, 145.1, 138.9, 133.6, 133.1, 131.5, 131.2, 127.9, 126.5, 121.8, 121.3, 121.0, 109.6, 109.1, 106.2, 102.8, 58.0, 55.9, 40.0, 32.5, 31.0(3), 26.8; HRMS: calcd. for C_28_H_28_N_3_O_5_Cl [M − Cl]^+^ 486.2023, found 486.2027.

*2,3-Methylenedioxy-9-((p-nitrophenylsulfonyl)oxy)-10-methoxy* *protoberberine chloride* (**3a**). Compound **1** (100 mg, 0.28 mmol) was treated with 4-nitrobenzenesulfonyl chloride (75 mg, 0.34 mmol) according to the general procedure to give the desired product **3a** as an orange solid, yield: 31%; M.p.: 146–148 °C (Dec.); ^1^H-NMR (500 MHz, DMSO-*d*_6_) δ 9.69 (s, 1H, CH_arom_), 9.14 (s, 1H, CH_arom_), 8.52 (d, *J* = 8.6 Hz, 2H, 2 × CH_arom_), 8.30 (d, *J* = 9.3 Hz, 1H, CH_arom_), 8.27 (d, *J* = 8.6 Hz, 2H, 2 × CH_arom_), 8.22 (d, *J* = 9.3 Hz, 1H, CH_arom_), 7.83 (s, 1H, CH_arom_), 7.13 (s, 1H, CH_arom_), 6.20 (s, 2H, OCH_2_O), 4.98 (t, *J* = 6.3 Hz, 2H, CH_2_), 3.69 (s, 3H, OCH_3_), 3.22 (t, *J* = 6.3 Hz, 2H, CH_2_); ^13^C-NMR (126 MHz, DMSO-*d*_6_) δ 151.8, 151.8, 150.7, 148.2, 144.2, 140.4, 139.3, 133.9, 131.6, 131.1, 130.8(2), 129.3, 126.5, 125.4(2), 122.1, 121.5, 120.6, 108.9, 106.1, 102.7, 57.3, 56.1, 26.6; HRMS: calcd. for C_25_H_19_N_2_O_8_SCl [M − Cl]^+^ 507.0856, found 507.0860.

*2,3-Methylenedioxy-9-((p-trifluoromethylphenyl sulfonyl)oxy)-10-methoxy* *protoberberine chloride* (**3b**). Compound **1** (100 mg, 0.28 mmol) was treated with 4-(trifluoromethyl)benzene-1-sulfonyl chloride (83 mg, 0.34 mmol) according to the general procedure to give the desired product **3b** as a yellow solid, yield: 41%; M.p.: 190–191 °C (Dec.); ^1^H-NMR (500 MHz, DMSO-*d*_6_) δ 9.70 (s, 1H, CH_arom_), 9.17 (s, 1H, CH_arom_), 8.30 (d, *J* = 9.2 Hz, 1H, CH_arom_), 8.24–8.20 (m, 4H, 4 × CH_arom_), 8.15 (d, *J* = 8.5 Hz, 2H, 2 × CH_arom_), 7.84 (s, 1H, CH_arom_), 7.13 (s, 1H, CH_arom_), 6.20 (s, 2H, OCH_2_O), 4.98 (t, *J* = 6.3 Hz, 2H, CH_2_), 3.23 (t, *J* = 6.3 Hz, 2H, CH_2_); ^13^C-NMR (126 MHz, DMSO-*d*_6_) δ 152.0, 150.9, 148.4, 144.5, 139.5, 139.2, 135.3, 134.1, 131.7, 131.3, 130.4(2), 129.5, 127.6(2), 126.6, 123.8, 122.3, 121.7, 120.8, 109.1, 106.3, 102.9, 57.4, 56.3, 26.8; HRMS: calcd. for C_26_H_19_F_3_NO_6_SCl [M − Cl]^+^ 530.0879, found 530.0884.

*2,3-Methylenedioxy-9-((m-trifluoromethylphenylsulfonyl)oxy)-10-methoxy* *protoberberine chloride* (**3c**). Compound **1** (100 mg, 0.28 mmol) was treated with 3-(trifluoromethyl)benzenesulfonyl chloride (55 µL, 0.34 mmol) according to the general procedure to give the desired product **3c** as an orange solid, yield: 37%; M.p.: 184–186 °C (Dec.); ^1^H-NMR (500 MHz, DMSO-*d*_6_) δ 9.71 (s, 1H, CH_arom_), 9.18 (s, 1H, CH_arom_), 8.31–8.26 (m, 2H, 2 × CH_arom_), 8.17 (d, *J* = 9.4 Hz, 1H, CH_arom_), 8.13 (t, *J* = 7.7 Hz, 1H, CH_arom_), 8.07–8.03 (m, 1H, CH_arom_), 7.99–7.94 (m, 1H, CH_arom_), 7.13 (s, 1H, CH_arom_), 6.20 (s, 2H, OCH_2_O), 5.04 (t, *J* = 6.4 Hz, 2H, CH_2_), 3.46 (s, 3H, OCH_3_), 3.24 (t, *J* = 6.4 Hz, 2H, CH_2_); ^13^C-NMR (126 MHz, DMSO-*d*_6_) δ 151.5, 150.7, 148.2, 144.2, 139.3, 136.4, 134.0, 133.8, 133.9, 133.0, 131.6, 131.4, 129.7, 129.3, 127.8, 126.3, 122.9, 122.4, 121.6, 120.6, 108.9, 106.1, 102.7, 57.2, 56.2, 26.6; HRMS: calcd. for C_26_H_19_F_3_NO_6_SCl [M − Cl]^+^ 530.0879, found 530.0883.

*2,3-Methylenedioxy-9-((o-trifluoromethylphenylsulfonyl)oxy)-10-methoxy* *protoberberine chloride* (**3d**). Compound **1** (100 mg, 0.28 mmol) was treated with 2-(trifluoromethyl)benzenesulfonyl chloride (52 µL, 0.34 mmol) according to the general procedure to give the desired product **3d** as an orange solid, yield: 38%; M.p.: 183–185 °C (Dec.); ^1^H-NMR (500 MHz, DMSO-*d*_6_) δ 9.73 (s, 1H, CH_arom_), 9.17 (s, 1H, CH_arom_), 8.36–8.29 (m, 3H, 3 × CH_arom_), 8.24 (d, *J* = 1.8 Hz, 1H, CH_arom_), 8.23 (d, *J* = 9.4 Hz, 1H, CH_arom_), 8.01 (t, *J* = 7.9 Hz, 1H, CH_arom_), 7.84 (s, 1H, CH_arom_), 7.13 (s, 1H, CH_arom_), 6.20 (s, 2H, OCH_2_O), 4.98 (t, *J* = 6.3 Hz, 2H, CH_2_), 3.66 (s, 3H, OCH_3_), 3.23 (t, *J* = 6.3 Hz, 2H, CH_2_); ^13^C-NMR (126 MHz, DMSO-*d*_6_) δ 151.8, 150.7, 148.2, 144.4, 139.2, 136.3, 134.0, 133.2, 132.8, 132.0, 131.5, 131.1, 130.7, 129.3, 126.3, 125.6, 123.6, 122.2, 121.5, 120.6, 108.9, 106.1, 102.7, 57.3, 56.1, 26.6; HRMS: calcd. for C_26_H_19_F_3_NO_6_SCl [M − Cl]^+^ 530.0879, found 530.0880.

*2,3-Methylenedioxy-9-((D-(+)-10′-camphorsulfonyl)oxy)-10-methoxy* *protoberberine chloride* (**3e**). Compound **1** (100 mg, 0.28 mmol) was treated with *D*(+)-10-camphorsulfonyl chloride (85 mg, 0.34mmol) according to the general procedure to give the desired product **3e** as a yellow solid, yield: 20%; M.p.: 193–195 °C (Dec.); ^1^H-NMR (500 MHz, DMSO-*d*_6_) δ 9.77 (s, 1H, CH_arom_), 9.14 (s, 1H, CH_arom_), 8.37 (d, *J* = 9.2 Hz, 1H, CH_arom_), 8.29 (d, *J* = 9.2 Hz, 1H, CH_arom_), 7.84 (s, 1H, CH_arom_), 7.12 (s, 1H, CH_arom_), 6.20 (s, 2H, OCH_2_O), 5.01 (t, *J* = 6.4 Hz, 2H, CH_2_), 4.20 (d, *J* = 15.0 Hz, 1H ,COCH_2_), 4.13 (s, 3H, OCH_2_), 3.98 (d, *J* = 15.0 Hz, 1H, COCH_2_), 3.23 (t, *J* = 6.4 Hz, 2H, CH_2_), 2.48–2.40 (m, 1H, CH_2_), 2.39–2.30 (m, 1H, CH_2_), 2.14 (t, *J* = 4.5 Hz, 1H, CH), 2.07–1.98 (m, 2H, CH_2_), 1.73–1.64 (m, 1H, CH_2_), 1.53–1.44 (m, 1H, CH_2_), 1.10 (s, 3H, CH_3_), 0.91 (s, 3H, CH_3_); ^13^C-NMR (126 MHz, DMSO-*d*_6_) δ 214.2, 152.3, 150.8, 148.4, 144.8, 139.1, 134.0, 132.1, 131.7, 128.6, 127.1, 122.5, 121.5, 120.8, 109.1, 106.2, 102.8, 58.5, 58.1, 56.4, 50.6, 48.8, 42.9, 42.6, 26.9, 26.8, 25.9, 20.0, 19.9; HRMS: calcd. for C_29_H_30_NO_7_SCl [M − Cl]^+^ 536.1737, found 536.1745.

*2,3-Methylenedioxy-9-((L-(−)-10′-camphorsulfonyl)oxy)-10-methoxy* *protoberberine chloride* (**3f**). Compound **1** (100 mg, 0.28 mmol) was treated with *L*(−)-10-camphorsulfonyl chloride (85 mg, 0.34 mmol) according to the general procedure to give the desired product **3f** as a yellow solid, yield: 21%; M.p.: 201–203 °C (Dec.); ^1^H-NMR (500 MHz, DMSO-*d*_6_) δ 9.76 (s, 1H, CH_arom_), 9.13 (s, 1H, CH_arom_), 8.37 (d, *J* = 9.2 Hz, 1H, CH_arom_), 8.29 (d, *J* = 9.2 Hz, 1H, CH_arom_), 7.84 (s, 1H, CH_arom_), 7.12 (s, 1H, CH_arom_), 6.20 (s, 2H, OCH_2_O), 5.01 (t, *J* = 6.4 Hz, 2H, CH_2_), 4.20 (d, *J* = 15.0 Hz, 1H, COCH_2_), 3.97 (d, *J* = 15.0 Hz, 1H, COCH_2_), 3.23 (t, *J* = 6.4 Hz, 2H, CH_2_), 2.47–2.40 (m, 1H, CH_2_), 2.38–2.31 (m, 1H, CH_2_), 2.14 (t, *J* = 4.5 Hz, 1H, CH), 2.06–1.98 (m, 2H, CH_2_), 1.73–1.64 (m, 1H, CH_2_), 1.53–1.46 (m, 1H, CH_2_), 1.10 (s, 3H), 0.91 (s, 3H); ^13^C-NMR (126 MHz, DMSO-*d*_6_) δ 214.2, 152.3, 150.8, 148.4, 144.8, 139.3, 134.0, 132.1, 131.7, 128.6, 127.9, 122.5, 121.5, 120.8, 109.1, 106.2, 102.8, 58.5, 58.1, 56.4, 50.6, 48.8, 42.9, 42.6, 26.9, 26.8, 25.9, 20.0, 19.9; HRMS: calcd. for C_29_H_30_NO_7_SCl [M − Cl]^+^ 536.1737, found 536.1742.

#### 3.2.2. Synthesis of 2,3-Methylenedioxy-9-(*o*,*p*-dimethoxybenzyl amino)-10-methoxy Protoberberine Chloride (**4**)

To a stirred solution of BBR (7.4 g, 20 mmol) in 2,4-dimethoxybenzylamine (15 mL, 78 mmol) at 120 °C for 6–8 h. The mixture was cooled to room temperature and washed with acetone (3 × 50 mL) to remove the remaining amine. The reside was purified by flash chromatography over silica gel using CH_2_Cl_2_/CH_3_OH (96.5:3.5) as the gradient eluent to afford red solid **4**, yield: 37%; M.p.: 239–240 °C (Dec.); ^1^H-NMR (500 MHz, DMSO-*d*_6_) δ 9.98 (s, 1H, CH_arom_), 8.73 (s, 1H, CH_arom_), 7.88 (d, *J* = 8.8, 1H, CH_arom_), 7.77 (s, 1H, CH_arom_), 7.52 (d, *J* = 8.8 Hz, 1H, CH_arom_), 7.14 (d, *J* = 8.3 Hz, 1H, CH_arom_), 7.09 (s, 1H, CH_arom_), 6.52 (d, *J* = 2.2 Hz, 1H, CH_arom_), 6.44 (t, *J* = 6.5 Hz, 1H, NH), 6.42–6.39 (m, 1H, CH_arom_), 6.17 (s, 2H, OCH_2_O), 4.80 (t, *J* = 6.2 Hz, 2H ,NCH_2_), 4.66 (d, *J* = 6.3 Hz, 2H, CH_2_), 3.87 (s, 3H, OCH_3_), 3.77 (s, 3H, OCH_3_), 3.71 (s, 3H, OCH_3_), 3.21 (t, *J* = 6.3 Hz, 2H, CH_2_); ^13^C-NMR (126 MHz, DMSO-*d*_6_) δ 160.5, 158.5, 150.1, 148.2, 148.1, 147.1, 137.5, 136.3, 133.5, 130.9, 130.3, 124.8, 121.2, 120.5, 120.3, 118.0, 117.7, 109.1, 105.9, 104.8, 102.6, 98.9, 57.5, 56.0, 55.8, 55.6, 47.0, 27.3; HRMS: calcd. for C_28_H_27_N_2_O_5_Cl [M − Cl]^+^ 471.1914, found 471.1919.

#### 3.2.3. Synthesis of 2,3-Methylenedioxy-9-amino-10-methoxy Protoberberine Chloride (**5**)

Compound **4** (3 g, 6.4 mmol) was dissolved in CH_3_OH, and hydrochloric acid 3 mL was added. The mixture was stirred for 5–6 h, filtered, and washed with 80% ethanol to afford red solid **5**, yield: 80%; M.p.: 212–214 °C (Dec.); ^1^H-NMR (500 MHz, DMSO-*d*_6_) δ 10.19 (s, 1H, CH_arom_), 8.64 (s, 1H, CH_arom_), 7.84 (d, *J* = 8.6 Hz, 1H, CH_arom_), 7.76 (s, 1H, CH_arom_), 7.32 (d, *J* = 8.6 Hz, 1H, CH_arom_), 7.08 (s, 1H, CH_arom_), 6.89 (s, 2H, NH_2_), 6.16 (s, 2H, OCH_2_O), 4.70 (t, *J* = 6.3 Hz, 2H, CH_2_), 3.98 (s, 3H, OCH_3_), 3.20 (t, *J* = 6.3 Hz, 2H, CH_2_); ^13^C-NMR (126 MHz, DMSO-*d*_6_) δ 149.8, 148.0, 147.0, 143.9, 138.1, 135.4, 132.1, 130.4, 123.0, 121.2, 119.8, 113.7, 113.3, 108.9, 105.6, 102.4, 56.9, 55.1, 27.2; HRMS: calcd. for C_19_H_17_N_2_O_3_Cl [M − Cl]^+^ 321.1233, found 321.1235.

#### 3.2.4. Synthesis of 2,3-Methylenedioxy-9-(2′-propylpentanamido)-10-methoxy Protoberberine Chloride (**6**)

To a stirred solution of **5** (100 mg, 0.28 mmol) and pyridine (100 µL, 1.24 mmol) in anhydrous CH_2_Cl_2_ (5 mL), the 2-propylpentanoyl chloride (143 µL, 0.84 mmol) was added and refluxed for 10–12 h. The solvent was removed by evaporation and purified by flash chromatography over silica gel using CH_2_Cl_2_/CH_3_OH (95:5) as the gradient eluent to give yellow solid **6**, yield: 34%; M.p.: 249–251 °C (Dec.); ^1^H-NMR (600 MHz, DMSO-*d*_6_) δ 10.04 (s, 1H, CH_arom_), 9.26 (s, 1H, CH_arom_), 8.99 (s, 1H, CH_arom_), 8.25–8.13 (m, 2H, CH_arom_), 7.79 (s, 1H, CH_arom_), 7.09 (s, 1H, CH_arom_), 6.15 (s, 2H, CH_arom_), 4.87 (t, *J* = 6.4 Hz, 2H, OCH_2_O), 3.99 (s, 3H, OCH_3_), 3.19 (t, *J* = 6.4 Hz, 2H, CH_2_), 2.67 (s, 1H, CH), 1.70–1.59 (m, 2H, CH_2_), 1.48–1.36 (m, 6H, 3 × CH_2_), 0.99 – 0.90 (m, 6H, 2 × CH_3_); ^13^C-NMR (151 MHz, DMSO-*d*_6_) δ 176.0, 154.8, 150.3, 148.1, 146.1, 137.8, 133.7, 131.0, 127.8, 125.8, 124.2, 122.3, 121.2, 120.8, 108.9, 106.0, 102.5, 57.4, 56.4, 45.6, 34.9(2), 26.8, 20.6(2), 14.6(2); HRMS: calcd. for C_27_H_31_N_2_O_4_Cl [M − Cl]^+^ 447.2278, found 447.2284.

#### 3.2.5. Synthesis of 2,3-Methylenedioxy-9-(2′-ethylhexyl amino)-10-methoxy Protoberberine Chloride (**7**)

To a stirred solution of BBR (2.5 g, 6.7 mmol) in 2,4-dimethoxybenzylamine (5 mL, 26 mmol) at 120 °C for 6–8 h. The mixture was cooled to room temperature and washed with acetone (3 × 20 mL) to remove the remaining amine. The reside was purified by flash chromatography over silica gel using CH_2_Cl_2_/CH_3_OH (95:5) as the gradient eluent to afford red solid **7**, yield: 41%; M.p.: 219–211 °C (Dec.); ^1^H-NMR (500 MHz, DMSO-*d*_6_) δ 10.29 (s, 1H, CH_arom_), 8.69 (s, 1H, CH_arom_), 7.88 (d, *J* = 8.7 Hz, 1H, CH_arom_), 7.76 (s, 1H, CH_arom_), 7.44 (d, *J* = 8.7 Hz, 1H, CH_arom_), 7.07 (s, 1H, CH_arom_), 6.54 (t, *J* = 6.0 Hz, 1H, NH), 6.16 (s, 2H, OCH_2_O), 4.82 (t, *J* = 6.3 Hz, 2H, CH_2_), 3.95 (s, 3H, OCH_3_), 3.50 (t, *J* = 6.4 Hz, 2H, NCH_2_), 3.20 (t, *J* = 6.3 Hz, 2H, CH_2_), 1.70–1.62 (m, 1H, CH), 1.45–1.15 (m, 7H, 2 × CH_2_ and CH_3_), 0.89–0.80 (m, 7H, 2 × CH_2_ and CH_3_); ^13^C-NMR (126 MHz, DMSO-*d*_6_) δ 150.1, 148.2, 147.0, 146.9, 138.1, 136.1, 133.6, 130.8, 124.9, 121.2, 120.2, 117.2, 116.2, 109.0, 105.8, 102.6, 57.4, 55.2, 51.0, 40.4, 30.9, 28.9, 27.3, 24.2, 23.2, 14.6, 11.3; HRMS: calcd. for C_27_H_33_N_2_O_3_Cl [M − Cl]^+^ 433.2485, found 433.2489.

#### 3.2.6. Synthesis of 2,3-Methylenedioxy-9-methoxy-10-(adamantane-1′-carbonyl)oxy Protoberberine Chloride (**16a**)

Compound **15** was obtained according to the reported procedure as an orange solid [18]. Compound **15** (100 mg, 0.28 mmol) was treated with 1-adamantanecarbonyl chloride (61 mg, 0.31 mmol) according to the general procedure to give the desired product **16a** as a brown solid, yield: 27%; M.p.: 144–146 °C (Dec.); ^1^H-NMR (500 MHz, DMSO-*d*_6_) δ 10.03 (s, 1H, CH_arom_), 9.07 (s, 1H, CH_arom_), 7.98 (d, *J* = 9.1 Hz, 1H, CH_arom_), 7.96 (d, *J* = 9.1 Hz, 1H, CH_arom_), 7.86 (s, 1H, CH_arom_), 7.12 (s, 1H, CH_arom_), 6.20 (s, 2H, OCH_2_O), 4.97 (t, *J* = 6.3 Hz, 2H, CH_2_), 4.10 (s, 3H, OCH_3_), 3.23 (t, *J* = 6.3 Hz, 2H, CH_2_), 2.14–2.07 (m, 9H, 3 × CH and 3 × CH_2_), 1.80–1.74 (m, 6H, 3 × CH_2_); ^13^C-NMR (126 MHz, DMSO-*d*_6_) δ 175.5, 151.0, 148.9, 148.4, 146.9, 141.6, 140.5, 138.0, 135.4, 132.0, 123.8, 122.2, 121.0, 120.8, 109.1, 106.4, 102.9, 63.8, 55.8, 41.3, 38.8(3), 36.4(3), 27.9(3), 26.8; HRMS: calcd. for C_30_H_30_NO_5_Cl [M − Cl]^+^ 484.2118, found 484.2122.

#### 3.2.7. Synthesis of 2,3-Methylenedioxy-9-methoxy-10-(2′-(adamantan-1-yl)acetoxy) Protoberberine Chloride (**16b**)

Compound **15** (100 mg, 0.28 mmol) was treated with 2-(1-adamantyl)acetyl chloride (66 mg, 0.31 mmol) according to the general procedure to give the desired product **16b** as an orange solid, yield: 28%; M.p.: 141–143 °C (Dec.); ^1^H-NMR (500 MHz, CDCl_3_) δ 10.85 (s, 1H, CH_arom_), 8.32 (s, 1H, CH_arom_), 7.78 (d, *J* = 8.8 Hz, 1H, CH_arom_), 7.74 (d, *J* = 8.8 Hz, 1H, CH_arom_), 7.39 (s, 1H, CH_arom_), 6.85 (s, 1H, CH_arom_), 6.12 (s, 2H, OCH_2_O), 5.42 (t, *J* = 6.2 Hz, 2H, CH_2_), 4.38 (s, 3H, OCH_3_), 3.32 (d, *J* = 6.2 Hz, 2H, COCH_2_), 2.45 (s, 2H, CH_2_), 2.09–2.04 (m, 3H, 3 × CH), 1.79 (d, *J* = 2.8 Hz, 6H, 3 × CH_2_), 1.78–1.70 (m, 6H, 3 × CH_2_); ^13^C-NMR (126 MHz, CDCl_3_) δ 169.4 151.4, 150.0, 148.7, 148.3, 141.6, 140.2, 137.7, 135.3, 131.5, 122.8, 122.1, 120.0, 119.5, 108.9, 105.5, 102.5, 64.2, 55.9, 48.5, 42.5(3), 36.8(3), 33.6, 28.7(3), 27.7; HRMS: calcd. for C_31_H_32_NO_5_Cl [M − Cl]^+^ 498.2275, found 498.2275.

### 3.3. Biology Assay

#### 3.3.1. Cell Culture and NF-κB Luciferase Reporter Gene Assay

The 293T cell line was purchased from the American Type Culture Collection (ATCC, VA, USA). Cells were cultured in Dulbecco’s Modified Eagle’s medium (DMEM) (Gibico, NY, USA) supplemented with 10% fetal bovine serum (Hyclone, UT, USA), 100 U/mL penicillin, and 100 μg/mL streptomycin sulfate and incubated at 37 °C in a humidified atmosphere with 5% CO_2_.

The 293T cells were co-transfected with pNF-κB-Lucreporter plasmid plus the pRL-TK plasmid using the Vigofect transfection reagent (Vigorous Biotechnology, Beijing, China) as instructed by the manufacturers [23]. After 24 h of transfection, cells were pretreated with 5 μM of BBR analogues for 2 h and then stimulated with TNF-α (20 ng/mL) for 6 h. Following TNF-α treatment, the cells were lysed, and the luciferase activity was determined using the Dual-luciferase reporter assay system (Promega, Madison, CA, USA) according to the manufacture′s protocols. The luciferase activity values were normalized to the expression of the Renilla luciferase and presented as the percentages of luciferase activity measured without TNF-α or BBR analogues treatment.

#### 3.3.2. Western Blot

Western blot was performed as described previously. Briefly, cells were washed with phosphate-buffered saline (PBS) and were lysed in lysis buffer (20 mM Tris-HCl, pH 7.5, 150 mM NaCl, 10 mM β-glycerophosphate, 5 mM egtazic acid (EGTA), 1 mM sodium pyrophosphate, 5 mM NaF, 1 mM Na_3_VO_4_, 0.5% Triton X-100, and 1 mM dithiothreitol (DTT)) supplemented with protease inhibitors (1 mM phenylmethylsulfonyl fluoride (PMSF), 5 μg/mL leupeptin, 5 μg/mL pepstatin A, and 5 μg/mL aprotinin). Proteins were separated by SDS-PAGE and were electrically transferred to a polyvinylidene difluoride membrane. The membranes were blocked for one hour at room temperature in TBS (150 mM NaCl, 20 Mm Tris, pH 7.5, 0.05% Tween-20) containing 5% milk and probed with specific first antibodies for one hour at room temperature. Blots were then incubated with horseradish peroxidase (HRP)-conjugated anti-rabbit (7074, Cell Signaling) or anti-mouse (7076, Cell Signaling) secondary antibody. Immunoreactive proteins were visualized using the ECL detection system (Bio-Rad, Hercules, CA, USA).

#### 3.3.3. Determination of Cytokines Production

The 293T cells cultured in 24-well plates were pretreated with compounds **2d**, **2e**, **2i** and **2j** for 2 h and then stimulated with TNF-α (20 ng/mL) for 6 h. Levels of IL-6 and IL-8 in the culture media were quantified using ELISA kits (R&D Systems Inc., MN, USA) in accordance with the manufacturer's instructions.

#### 3.3.4. Cell Survival Assay

The cell survival was evaluated by MTT assay. Briefly, Cell suspensions (100 μL) of 293T cells at concentration of 50% confluence were seeded into the 96-well plates, and then were treated with various concentrations of BBR derivate. After 24 h of incubation, 10 μL of the MTT (1 mg/mL) solution was added into each plate and incubated for 2 h at 37 °C, 5% CO_2_. Subsequently, the culture supernatant was replaced with 100 μL DMSO to dissolve the formazan crystal made from succinic dehydrogenase in the mitochondria and its substrate MTT. The optical density (OD) at 550 and 630 nm were measured using a microplate reader. The net absorbance (OD630–OD550) indicates the enzymatic activity of mitochondria and provides information on cell viability.

## 4. Conclusions

To conclude, 23 new BBR analogues defined on substituents of the ring D were synthesized and evaluated for their effect to inhibit TNF-α-induced NF-κB activation. SAR analysis indicated that tertiary or quaternary carbon substitutions on position 9 or rigid fragment on position 10 might be beneficial for the activity. Among them, compounds **2d**, **2e**, **2i** and **2j** exhibited satisfactory potency with inhibitory rates of over 90% at the concentration of 5 μM compared with that of BBR. A preliminary mechanism study revealed that all of them could inhibit TNF-α-induced NF-κB activation via impairing IKK phosphorylation as well as TNF-α induced expression of cytokines including IL-6, and IL-8. Our current study supports the potential role of compounds **2d**, **2e**, **2i** and **2j** in the prevention and treatment of inflammatory diseases, and they have been selected for the further investigation.

## Figures and Tables

**Table 1:**
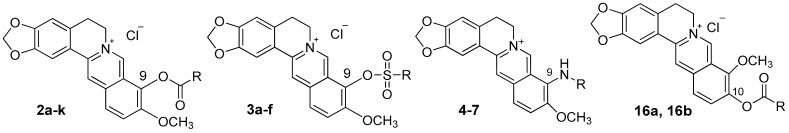
Structures and inhibition effect of target compounds on tumor necrosis factor (TNF)-induced nuclear factor (NF)-κB activities.

No.	R	Inhibitory Rate%	No.	R	Inhibitory Rate%	No.	R	Inhibitory Rate%
**2a**	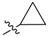	56	**2i**	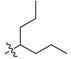	96	**3f**	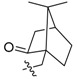	48
**2b**	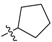	34	**2j**	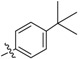	88	**4**	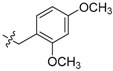	43
**2c**	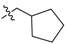	52	**2k**	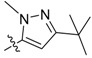	72	**5**	H	52
**2d**	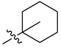	96	**3a**	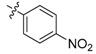	65	**6**	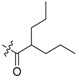	43
**2e**	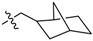	83	**3b**	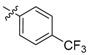	86	**7**	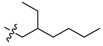	37
**2f**	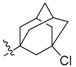	39	**3c**	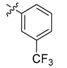	85	**16a**	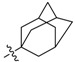	96
**2g**	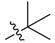	81	**3d**	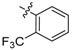	87	**16b**	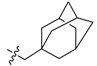	84
**2h**	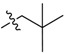	92	**3e**	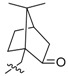	82	BBR	-	36
						PS1145	-	73

**Table 2:** The IC_50_ of compounds **2d**, **2e**, **2i** and **2j** on inhibition of TNF-α-induced NF-κB activation.

Compound	2d	2e	2i	2j	PS1145
IC_50_ (ìM)	1.01 ± 0.19	3.91 ± 1.04	0.55 ± 0.43	10.13 ± 1.40	2.493 ± 0.23

## Supplementary Materials

Supplementary File 1
