# Supplementary material for: Synthesis and Identification of Novel Berberine Derivatives as Potent Inhibitors against TNF-α-Induced NF-κB Activation
Source: Molecules. 2017 Jul 27;22(8):1257. doi: 10.3390/molecules22081257 (PMC6152030; doi:10.3390/molecules22081257)

Supporting Information

Article

Synthesis and Identification of Novel Berberine Derivatives as Potent Inhibitors against TNF-α-induced NF-κB Activation

Yan–Xiang Wang, Lu Liu, Qing–Xuan Zeng, Tian–Yun Fan, Jian–Dong Jiang, Hong–Bin Deng and Dan–Qing Song

Table of Contents

^{1}H-NMR and ^{13}C-NMR sprectrums of the representatives **2e**, **2g** and **2i** 2–4

BRUKER AV-III-500 ^{1}H-NMR **2e** IN DMSO

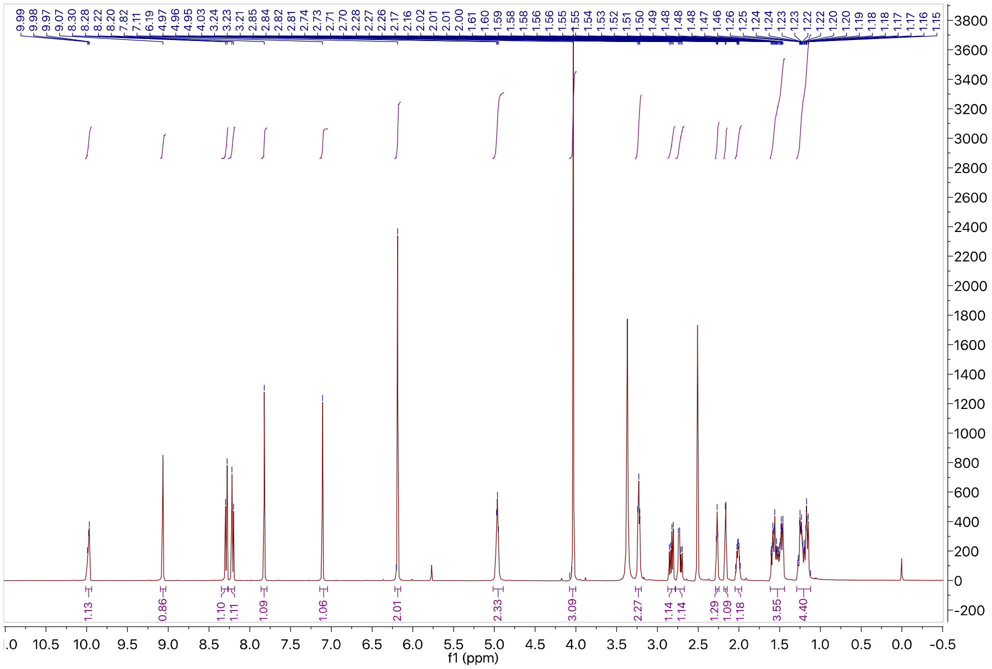

BRUKER AV-III-500 ^{13}C-NMR **2e** IN DMSO

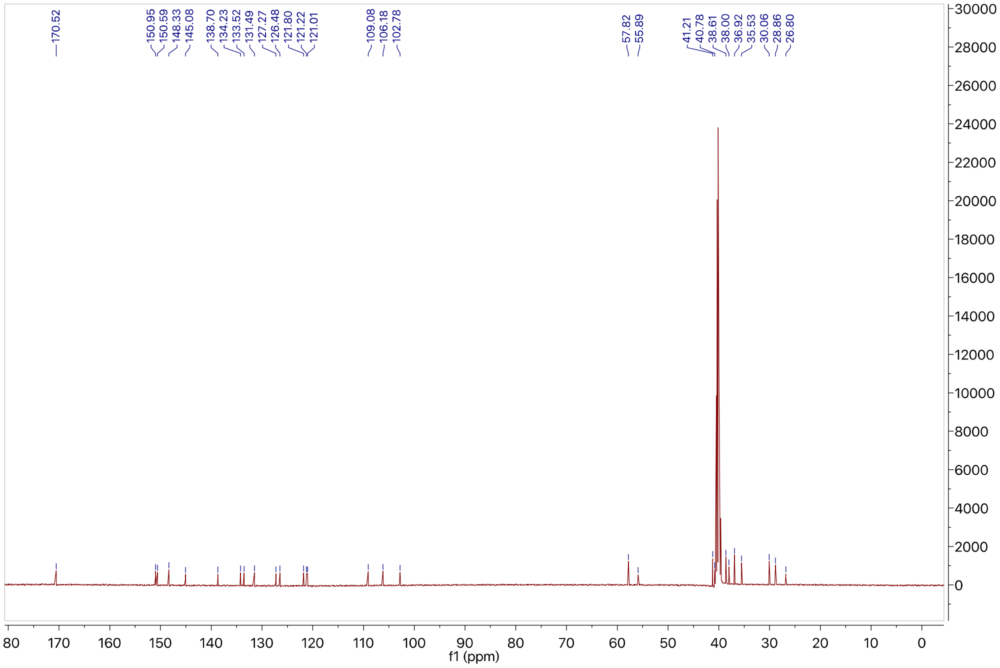

BRUKER AV-III-500 ¹H-NMR **2g** IN DMSO

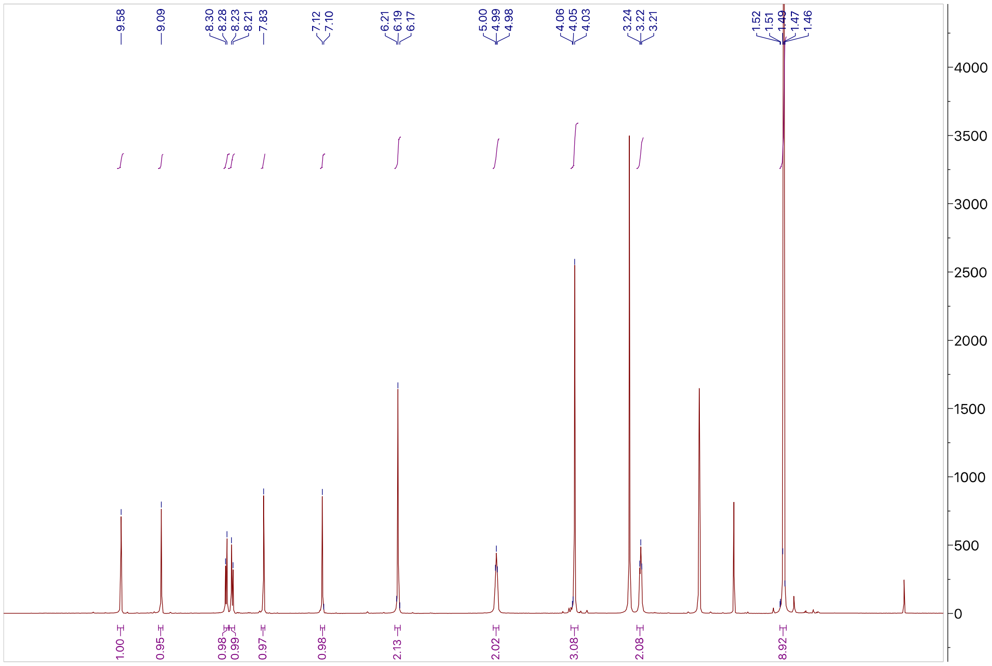

VNS-600 CARBON **2g** IN CD_3OD

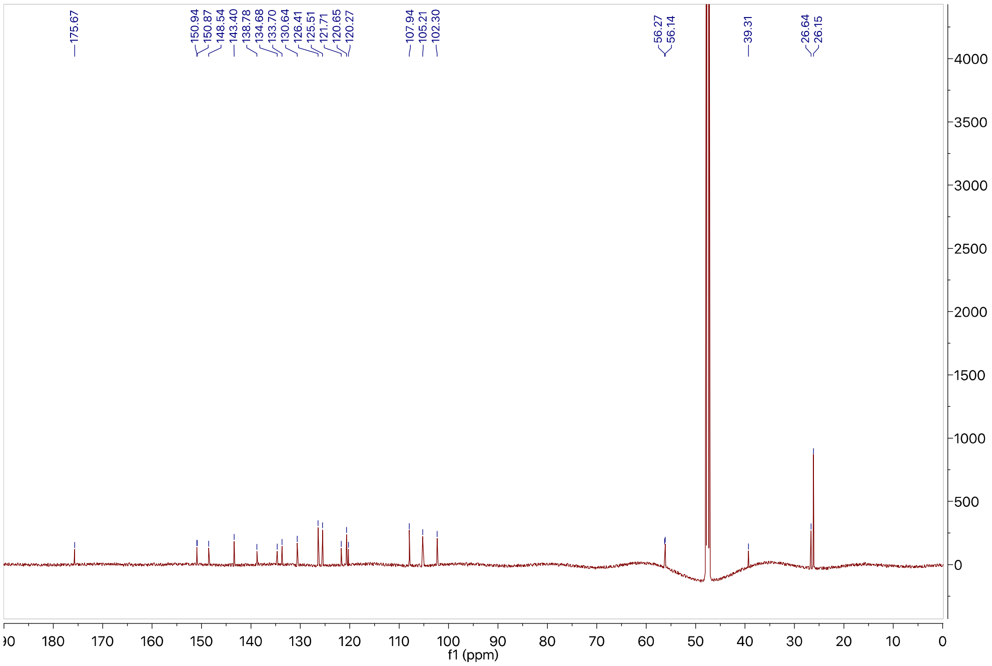

BRUKER AV-III-500 ¹H-NMR **2i** IN DMSO

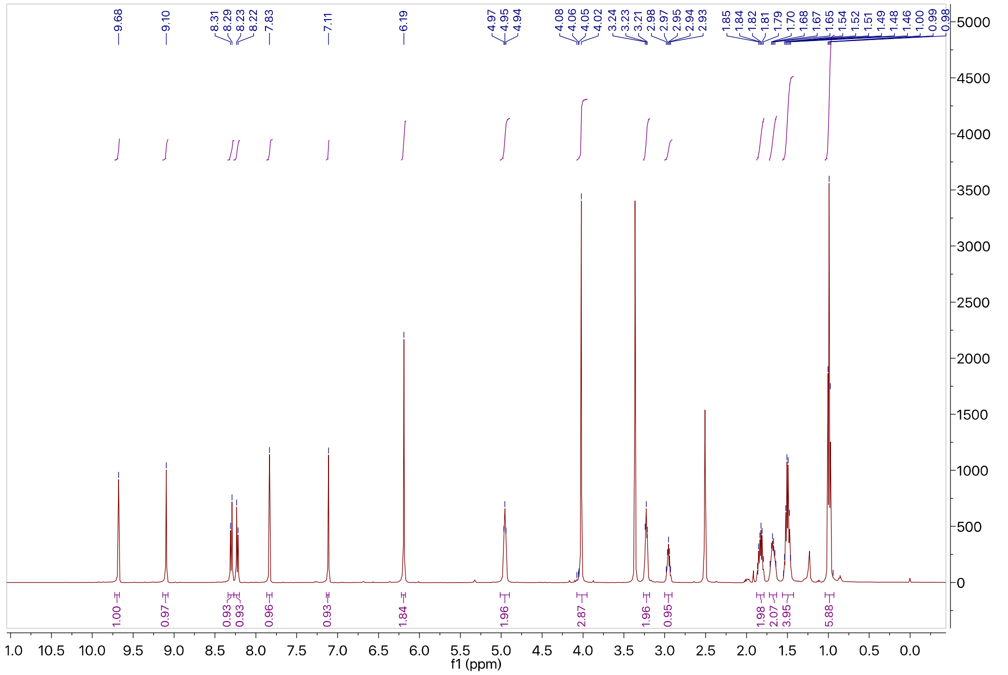

BRUKER AV-III-500 ¹³C-NMR **2i** DMSO

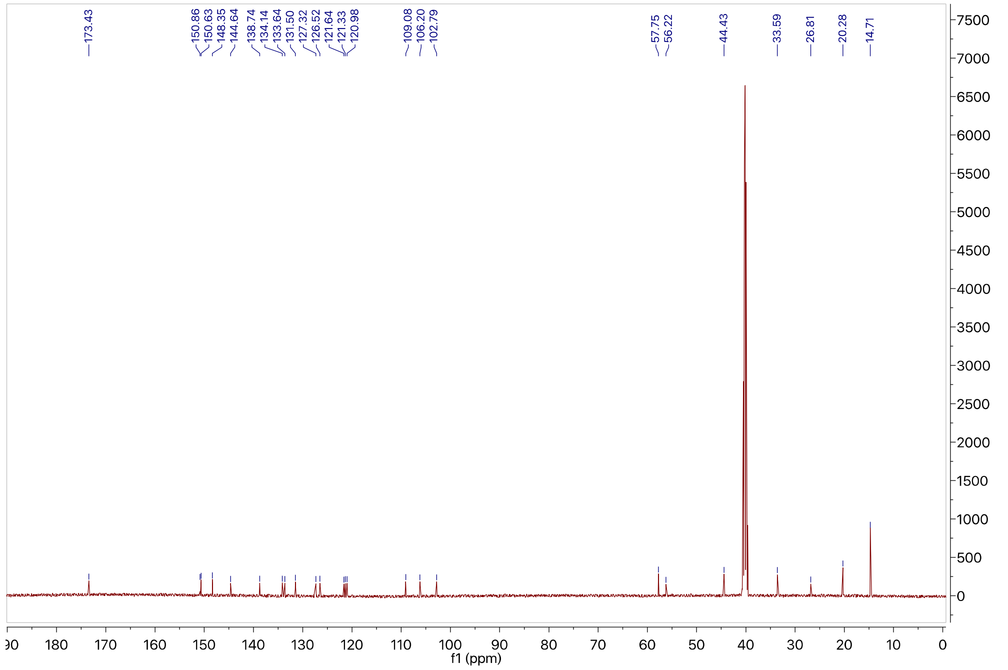

Supplement: Supplementary File 1 [file molecules-22-01257-s001.pdf]
